# The Role of MicroRNAs in Aβ Deposition and Tau Phosphorylation in Alzheimer’s Disease

**DOI:** 10.3389/fneur.2017.00342

**Published:** 2017-07-18

**Authors:** Juanjuan Zhao, Dongxu Yue, Ya Zhou, Li Jia, Hairong Wang, Mengmeng Guo, Hualin Xu, Chao Chen, Jidong Zhang, Lin Xu

**Affiliations:** ^1^Department of Immunology, Zunyi Medical College, Guizhou, China; ^2^Department of Medical Physics, Zunyi Medical College, Guizhou, China

**Keywords:** Alzheimer’s disease, microRNAs, amyloid-beta, amyloid precursor protein, Tau protein

## Abstract

Alzheimer’s disease (AD), with main clinical features of progressive impairment in cognitive and behavioral functions, is the most common degenerative disease of the central nervous system. Recent evidence showed that microRNAs (miRNAs) played important roles in the pathological progression of AD. In this article, we reviewed the promising role of miRNAs in both Aβ deposition and Tau phosphorylation, two key pathological characters in the pathological progression of AD, which might be helpful for the understanding of pathogenesis and the development of new strategies of clinical diagnosis and treatment of AD.

## Introduction

It is estimated worldwide that as many as 46.8 million people suffer from Alzheimer’s disease (AD) in 2015. By the year 2050, this number would further exceed 1.315 billion (1). AD is a kind degenerative disease of central nervous system, which is characterized by clinical features such as early stage memory loss, cognitive impairment, and personality change in the early and old age. The main pathological hallmark in brain of AD patients was β-amyloid (amyloid-β, Aβ) deposition-formed extracellular amyloid plaques (senile plaques) and intracellular neurofibrillary tangles, which were mainly composed by the excessive phosphorylation of Tau protein and loss of neurons. Despite the discovery of numerous molecular changes in AD, the pathogenesis of AD is still not entirely clear.

MicroRNAs (miRNAs) are a novel class of short (18–25 nucleotides) and single-stranded non-coding RNAs involved in the posttranscriptional regulation of gene expression. Their mechanism of action is mediated by complement binding to the 3’untranslated region (3’UTR) of mRNA, leading to degradation or translation repression of the target mRNA (2). In recent years, miRNAs have attracted increased research interest and a substantial amount of work has been reported that miRNAs could be used as new targets for diagnosis and treatment of a variety of neurodegenerative diseases (3, 4). Although researches that were focused on the role of miRNAs in AD have only just began (5), there have been already some evidence showing that aberrantly expressed miRNAs such as miR-29b (6), miR-399 (7), miR-106b (8), miR-34a (9), let-7 (10), miR-155 (11), and miR-7 (12) were present in different cell types at different stages of AD. These literatures indicated that multiple miRNAs might be contributed to the development of AD. Especially, functional studies have further shown that these miRNAs were closely related to the changes of both Aβ formation and Tau phosphorylation, which were vital in the pathogenesis of AD (13, 14). Therefore, these observations provided novel and important evidences for successive study on the investigation of underlying mechanism of AD and the development of new targets for clinical therapy.

## miRNAs and Aβ

### miRNAs and Amyloid Precursor Protein (APP)

Amyloid precursor protein, which is a source of Aβ, is widely present in all histiocytes and especially abundant in neurons and astrocytes. Posttranscriptional alternative splicing resulted in six APP isoforms. Among these isoforms, APP695 is the most dominant type in normal brain, whereas APP751 is found at a higher level in brain of AD patients. Recent studies have shown that some miRNA molecules were involved in the regulation of alternative splicing of APP. Donev et al. reported that neuronal cells transfected with APP695, which was the main expressed subtype of APP mRNA lacking exons 7 and 8, produced significantly less Aβ, than the sequence of full-length APP-transfected neuronal cells did (15). Studies by Smith et al. further showed that APP695 expression was noticeably downregulated and Aβ production significantly increased in neuronal cells from mice with knocked out of *Dicer* gene, which was deficient for a broad range of miRNAs (16). Successive work reported that the significantly decreased level of miR-124, a representative member of miRNA family, in the brain of AD patients, greatly improved the expression of its corresponding target molecule polypyrimidine tract-binding protein1 (PTBP1), which was critical splicing factor to interrupt the splicing process of APP exons 7 and 8 and subsequently accelerate Aβ generation and AD progression (17). Combining these data demonstrated that specific miRNAs may influence the formation of Aβ through regulating APP splicing.

Prior researches on the regulation of APP expression by miRNAs were mainly focused on direct targeted regulation. For instance, Niwa et al. found that, in *C. elegans*, the expression of APP homolog—*apl-1*, an important gene for developmental regulation, was regulated by miRNA family member *let-7* (17). In subsequent studies, it was found that APP was a direct target of some members of the miRNA family, such as miR-20a, miR-155, miR-17 and miR-106b (18, 19), miR-135 and miR-200b (20), miR-101 (21), miR-16 (22) and miR-147, miR-153, miR-323-3p, miR-644, and miR-655 (23, 24). Recent studies further revealed that miRNAs could affect the expression of APP by binding to APP 3’UTR *cis*-regulatory elements. For example, Rck/p54 was a component of a protein complex near the APP stop codon, and its main role was to enhance APP mRNA and protein expression level in neuronal cells through interacting with APP mRNA. Several new studies reveal that miRNAs may be involved in transcription and translation of APP by interacting with Rck/p54 (25, 26). Besides, other research work showed that 81-nucleotide *cis*-regulatory sequence on APP mRNA was not only the binding target for miR-106b/miR-520c but also consisted of a predicted-binding site for miR-20 family members. However, whether these elements were working in cooperative or competitive manners in the pathogenesis of AD remains to be investigated.

Interestingly, other studies further showed that a single nucleotide polymorphism (SNP) at the 3’UTR of APP mRNA could interfere with the binding of miRNAs to APP mRNA (24), indicating the complexity of regulation of miRNAs on APP expression. Among these SNPs, SNP T171C was found to significantly inhibit the binding of miR-147, which resulted in an increased expression of APP and subsequent generation of Aβ, whereas SNP A454G increased the binding of miR-20a to APP mRNA, thereby reducing APP expression. These data showed that SNPs were involved in the regulation of APP expression by miRNA and may, therefore, be potential risk factors for AD (27). In short, miRNAs could affect APP expression in numerous ways, such as by direct binding to APP 3’UTR, or by interacting with APP mRNA regulatory elements and AD-associated SNPs. However, the exact underlying mechanism remains to be fully elucidated.

### miRNAs and BACE1

As the rate-limiting enzyme for Aβ production, β-site APP cleaving enzyme (BACE1) has attracted increased interest in pathogenesis of AD. Numerous studies have found that BACE1 mRNA level were regulated by various miRNA molecules. For example, a recent study has shown that miR-485-5p could inhibit BACE1 translation through binding to BACE1 exon 6 and overexpression of miR-485-5p reduced BACE1 protein level by 30% (28). Kim et al. showed that miR-186 could inhibit BACE1 expression by direct binding its mRNA 3’UTR in neuronal cells, which suggest that miR-186 downregulated might be a risk factor for the development of AD (29). Meanwhile, Hebert et al. have also shown that upregulation of miR-29a, -29b-1, and -29b-9 levels could directly inhibit BACE1 expression in neuronal cells (30). Consistent with these findings, reduced level of miR-29a/b-1 in cultured neuronal cells *in vitro* significantly increased the expression of Aβ and vice versa. These research data suggested that the miR-29a/b family may potentially inhibit the expression of BACE1 protein level and, therefore, be related to the pathogenesis of AD. In addition, Lei et al. also showed that increased miR-29c expression *in vitro* directly reduced BACE1 protein level by binding to the 3’UTR of its mRNA (31). These results were consistent with the finding that the amount of brain miR-29c was doubled in BACE1-knockdown mice compared with that in wild-type mice. Moreover, Boissonneault et al. also confirmed that miR-298 and miR-328 regulated BACE1 protein expression in neuronal cells by targeting specific binding sites of the BACE1 3’UTR (32). Finally, during the pathogenesis of AD, BACE1 mRNA levels of neuronal cells negatively correlated with miR-107 level (33). Conversely, increased BACE1 level was accompanied with a decreased level of miR-107 (33). Bioinformatics and luciferase gene reporter assays further confirmed that miR-107 could effectively bind to the 3’UTR sequence of BACE1. In summary, the above data demonstrated that a number of miRNAs could control BACE1 translation by binding to the 3’UTR of BACE1 mRNA, which affected the modification and metabolic processes of the Aβ protein, reflecting the complexity of regulatory mechanisms involved in pathogenesis of AD.

### Additional Factors Affecting Aβ Production

It has been demonstrated that Aβ formation could be regulated by membrane ceramide, an important component of lipid rafts and was found at high level in sporadic AD. Moreover, ceramide could promote lipid raft formation via erroneous localization of BACE1 and γ-secretase, thereby increasing Aβ production (34). During that process, the first rate-limiting enzyme of ceramide synthesis was serine palmitoyltransferase (SPT). In a recent study by Geekiyanage and Chan, it was shown that the SPT protein was regulated by miR-181c, miR-137, miR-29a/b-1, and miR-9 (35). These miRNA molecules, which were abnormally downregulated in the AD frontal cortex, may modify the lipid composition and affect the level of Aβ formation through increasing SPF level. The expression of miR-181c, miR-137, and miR-29a/b-1, which were related to growth and development, reached peak levels in adult mice. Conversely, SPT expression negatively correlated with growth, i.e., the SPT level steadily decreased with age. The incidence of AD was higher in women than that in men (36), which also might be related to higher SPT protein level and lower miR-181c, -137, -29a/b-1 levels in the female population (35). Interestingly, it has been reported that a high-fat diet could increase plasma level of ceramides, thereby exacerbating the burden of Aβ deposition in animal models. In this experimental group, the expression levels of miR-181c and miR-137 were abnormally lower. These studies indicated that regulation of SPT by miRNAs provided a novel mechanism of pathogenesis of AD, which was also related to some AD-related risk factors such as age, gender, and intake of high-saturated fat.

Cholesterol homeostasis also has significant impact on Aβ metabolism and deposition. APP, BACE1, and PSEN1 are all membrane-bound proteins, whose hydrolysis and transportation are dependent on membrane fluidity, which is governed by cholesterol. Meanwhile, cholesterol can be transported out of cells by the ATP-binding cassette transporter A1 (ABCA1) to form high-density lipoprotein particles (37), which reduce the risk of AD (38). Recent research work further showed that ABCA1 deficiency in neurons could decrease the cellular cholesterol efflux and subsequently enhance Aβ deposition. Moreover, overexpression of ABCA1 significantly decreased Aβ accumulation (33). Similarly, ABCA1 expression level was found increased in the hippocampus of AD patients and was positively correlated to the severity of cognitive impairment (39). Interestingly, some new studies have demonstrated that, in neurons, miR-33 could directly inhibit the expression of ABCA1 by binding to the 3’UTR of ABCA1 mRNA, which reduced the efflux of cellular cholesterol and increased the level of Aβ, thereby increasing the risk of AD (33).

In summary, miRNAs could affect Aβ metabolism in many ways including through their direct action on 3’UTR of APP, BACE1, ABCA1, and other related genes, as well as indirect regulation through other factors, and played important roles in the pathogenesis of AD.

## miRNAs and Tau Protein

Microtubule-associated protein Tau (MAPT) is another major player in the pathogenesis of AD. The disruption of any of the steps in the Tau protein metabolic pathway may exacerbate the progression of AD. Recent studies have shown that conditional knockout of the Dicer gene in the mouse brain created an extensive miRNA deficiency, which resulted in abnormal Tau protein metabolism in mice with AD-like Tau hyperphosphorylation and aberrant splicing of MAPT (40, 41). Similar studies by Bilen et al. showed that when Dicer1 was knocked out, there were increased level of Tau protein and neurodegeneration in the Drosophila brain (42). These results indicated that miRNAs played key roles in the regulation of Tau protein metabolism and subsequently were involved in the pathogenesis of AD.

### miRNAs and Tau Protein Editing

Alternative splicing of exons 2, 3, and 10 has resulted in 6 Tau isoforms in the human brain (43). Exon 10 encodes the microtubule-binding repeat regions and is responsible for generating 3R or 4R isoforms. Under normal conditions, the ratio between 4R/3R isoforms is approximately 1:1 and an imbalance of this ratio could cause neurodegenerative diseases, such as dementia (44, 45). An important finding was reported by Smith et al. that many miRNAs, including miR-124, miR-9, miR-132, and miR-137, could affect the 4R/3R ratio in neuronal cells by regulating MAPT splicing (41). Further analysis of miRNA expression profiling of patients with progressive supranuclear palsy, a neurodegenerative disease caused by Tau 4R isoform overexpression, showed that miR-132 level were significantly downregulated in a patients’ brain, whereas the level of polypyrimidine tract-binding protein 2 (PTBP2), a target of miR-132, increased obviously. Importantly, overexpressing miR-132 or suppressing PTBP2 expression could reverse the ratio of 4R/3R isoforms (41). Similarly, *in vitro* experimental setting, PTBP1 was found closely related to MAPT exon 10 splicing (46). Moreover, its level was upregulated in conditional Dicer-knockout mouse brains (16). In addition, PTBP1/2 level were also significantly altered during the development of diseases associated with alternative splicing (47, 48). In summary, these data showed that specific miRNAs were involved in the regulation of MAPT exon 10 splicing and subsequently contributed to the pathogenesis of AD.

### miRNAs and Tau Protein-Associated Kinases

Dysregulation of the balance of protein kinases and phosphatases is the direct cause of Tau protein hyperphosphorylation. It is now known that Tau protein phosphorylation can be mediated by a variety of protein kinases, such as extracellular regulated protein kinases (ERKs) and glycogen synthase kinase-3 (GSK-3). In studies described earlier in this review, Hebert et al. conditionally knocked out Dicer enzyme gene in the mouse brain and found that there were hyperphosphorylation of endogenous Tau protein in pathological regions and increased expression of mitogen-activated protein kinase 3 (MAPK3/ERK1) and GSK-3β. Bioinformation analysis further showed that the 3’UTR of mRNA of these protein kinase molecules could be directly bound by some miRNA mature sequences (40). *In vitro* studies from this research group also showed that in murine neuronal cells, some members of the miR-15 family, including miR-15a, miR-16, miR-195, and miR-497, could directly interact with the 3’-UTR of ERK1 mRNA (40). Meanwhile, to another protein kinase GSK-3β, which could hyperphosphorylate the PHF1 site of the Tau protein and was associated with both Aβ generation and NFT formation in AD brain (49), Mohamed et al. recently reported that miR-26a could directly regulate GSK-3β gene expression (50). Finally, it would be noticed that other research work further suggested that miR-26a was also involved in the pathogenesis of the AD (19), which was related to its interaction with brain-derived neurotrophic factor, an important neurotrophin for neuronal development and plasticity (51), indicating the complexity of target molecules of distinct miRNA molecules in the development of AD. Collectedly, these literatures suggested that various miRNAs could blind directly to phosphorylated Tau-associated protein kinase molecules or indirectly regulate the level of associated protein kinase molecule, so as to form a complex biological regulatory network of miRNAs regulated phosphorylated Tau-related protein kinase. However, the exact role of different miRNAs in the pathogenesis of AD through the regulation of protein kinases remains to be illuminated.

### miRNAs and Tau Protein Clearance

Acetylation at specific sites of the Tau protein can promote its autophosphorylation, exacerbating abnormal Tau protein aggregation and facilitating AD progression. The Tau protein acetylation state is mainly dependent on acetyltransferase p300 (an acetylase) and sirtuin 1 (SIRT1, a deacetylase). With the reduced level of SIRT1 in the AD brain, the acetylation level of Tau protein aggravated correspondingly (52, 53), followed by significant accumulation of phosphorylated Tau protein (54). Recent studies have demonstrated that the SIRT1 gene could be directly inhibited by miR-9, miR-212, and miR-181c, respectively (55, 56). In addition, co-chaperone BCL2-associated athanogene 2 (BAG2) could preferentially and efficiently degrade insoluble and phosphorylated Tau, primarily through forming a complex with Hsp70 on microtubules by capturing and delivering Tau protein to the non-ubiquitin-independent proteasomal pathway for degradation. During this process, miR-128a, as an intrinsic regulator, could fine-tune tau protein level in neuronal cells by acting directly on 3’-UTR of BAG2 mRNA (57). Combing these literatures suggested that miRNAs may regulate the edition and clearance of Tau protein, as well as accumulation of phosphorylated Tau protein. However, whether Tau protein itself could induce the degradation of distinct miRNA molecules and subsequently affect the pathogenesis of AD remain to be further investigated.

## Summary

To date, some active advances have been made on the potential value of miRNAs expression on early detection of AD. For example, some recent studies showed that the levels of miR-126b and miR-27a were significantly reduced, whereas levels of miR-9, miR-125b, miR-15, and miR-138 were significantly increased in the cerebrospinal fluid of clinical AD patients (58, 59). Similarly, the serum levels of various other miRNAs, such as miR-15a, let-7d, let-7g, miR-142, miR-191, miR-301a, miR-545, and miR-342-3p in clinical AD patients were significantly different from those in normal population (54, 55). Moreover, other studies further showed that the expression of miR-34a and miR-181b were also significantly increased in peripheral mononuclear blood cells of AD patients (52). Importantly, it has been found that the altered expression of distinct miRNAs in AD patients appeared earlier than Aβ deposition and Tau phosphorylation, which were as biomarkers for early diagnosis of AD currently (16). These studies suggested that the altered expression of specific miRNAs might be used as important promising biomarkers for AD prediction and/or diagnosis.

**Figure 1:**
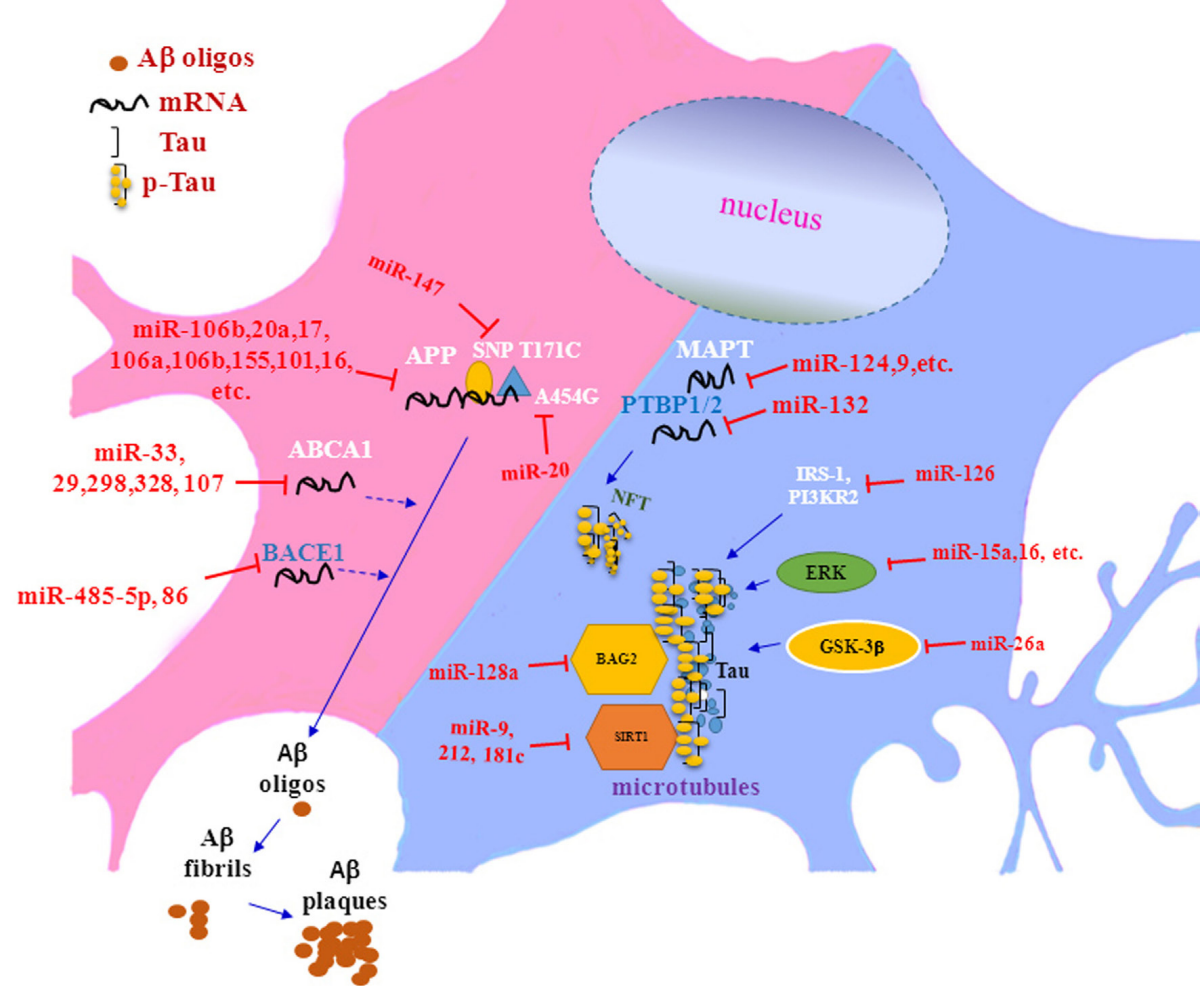
The role of microRNAs in Aβ deposition and tau phosphorylation in pathogenesis of Alzheimer’s disease.

In summary, recent researches have proven that miRNAs could be participated in the pathogenesis of AD by regulating multiple targets (see Table 1; Figure 1). However, there are still a large number of scientific questions worthy to further exploration. For example, although some studies have shown that adding exogenous Aβ42 peptides to rat hippocampal neurons could lead to rapid downregulation of multi-specific miRNAs (60), it was still a question whether or not abnormal expression of miRNAs was an accompanied phenomenon or/and induction factor for the pathogenesis of AD. Another question was how can a concerted subnetwork of potential target genes of miRNAs be set up, in which miRNAs specifically aimed to its target genes in AD. Brain degeneration in AD patients is a heterogeneous process, therefore, the pathological changed in some brain regions was earlier and more severe than that in other brain regions, making it more challenging for researchers to decipher the exact roles of miRNAs in the complex process. Together, further in-depth miRNA research and discovery will provide novel promising ideas and strategies for elucidating the pathological mechanisms of AD and for developing effective methods for early diagnosis and treatment of AD.

**Table 1:** microRNAs (miRNAs) in Alzheimer’s disease.

Dysregulated miRNA(s)	Level of brain tissue	Target site(s)	Reference
miR-124	Decreased	PTBP1/2	(17)
miR-106b, -20a, -17, -106b, -106a, -155, -101, -16, -147, -153, -323-3p, -644, -655	Decreased	APP	(18–24)
miR-485-5p, -29a, -29b-1, -9, -29c, -298, -328, -107	Decreased	BACE1	(28–33)
miR-9, -29a/b-1, -137, -181c	Decreased	SPT	(35)
miR-33	Decreased	ABCA1	(37)
miR-132	Decreased	PTBP2	(41)
miR-26a	Increased	GSK-3β	(50)
miR-9, -34, -181c	Increased	SIRT1	(55, 56)
miR-128a	Increased	BAG2	(57)
miR-139	Increased	CB2	(61)
miR-206-3p	Increased	BDNF	(62)
miR-181c	Decreased	crmp2	(63)
miR-212	Decreased	PTEN/FOXO3a	(64)
miR-218	Increased	PTPα	(65)
miR-125b	Increased	15-LOX	(66)
miR135a	Increased	THBS1	(67)

*PTBP1/2, polypyrimidine tract-binding protein1/2; APP, amyloid precursor protein; BACE1, β-site amyloid precursor protein cleaving enzyme; SPT, serine palmitoyltransferase; ABCA1, ATP-binding cassette transporter A1; GSK-3β, glycogen synthase kinase-3; SIRT1, silent mating type information regulation 2 homolog 1 or sirtuin 1; BAG2, BCL2-associated athanogene 2; CB2, cannabinoid receptor type 2; BDNF, brain-derived neurotrophic factor; crmp2, collapsin response mediator protein 2; PTEN, phosphatase and tensin homolog deleted on chromosome ten; FOXO3a, Forkhead box O3; PTPα, protein tyrosine phosphatase α; 15-LOX, 15-lipoxygenase; THBS1, thrombospondin 1*.

## Author Contributions

JZ, DY, YZ, LJ, HW, MG, HX, CC, and JZ carried out reading the related literatures and drafting the manuscript. LX and JZ designed the outline of manuscript. All authors read and approved the final manuscript.

## Conflict of Interest Statement

All authors declare that the research was conducted in the absence of any commercial or financial relationships that could be construed as a potential conflict of interest.
